# Diagnostic Accuracy of the Delta Neutrophil Index in Predicting Sepsis-Associated Acute Kidney Injury: A Cross-Sectional Study

**DOI:** 10.7759/cureus.114240

**Published:** 2026-08-09

**Authors:** Yallambhotla Varuneil, Shubhransu Patro, Nikunj Kishore Rout, Parmarth Arora, Nupur M Bhalerao, Jyoti Prakash Sahoo

**Affiliations:** 1 General Medicine, Kalinga Institute of Medical Sciences, Bhubaneswar, IND; 2 Nephrology, Kalinga Institute of Medical Sciences, Bhubaneswar, IND; 3 Pharmacology, Kalinga Institute of Medical Sciences, Bhubaneswar, IND

**Keywords:** biomarkers of acute kidney injury, c-reactive protein (crp), delta neutrophil index, diagnostic accuracy study, procalcitonin level, receiver operating characteristic (roc) analysis, renal dysfunction severity, sepsis markers, serum lactate, sofa score

## Abstract

Background and objectives: Sepsis-associated acute kidney injury (SA-AKI) is a worldwide health concern. It necessitates prompt and precise diagnosis of sepsis and renal dysfunction. The delta neutrophil index (DNI), C-reactive protein (CRP), procalcitonin, and serum lactate are potential biomarkers for early detection of SA-AKI. Hence, we conducted this study to evaluate the diagnostic accuracy of DNI in predicting SA-AKI among patients with sepsis and to calculate the area under the curve (AUC). We also compared the same with those of CRP, procalcitonin, and serum lactate. Materials and methods: This cross-sectional study was carried out at Kalinga Institute of Medical Sciences (KIMS), Bhubaneswar, India, from March 2024 to February 2026. We enrolled adult sepsis patients from the medicine intensive care unit (ICU) during this period. We excluded referred cases, patients with sepsis before ICU admission, and those with prior renal dysfunction. White blood cell (WBC) count, serum creatinine, estimated glomerular filtration rate (eGFR), CRP, procalcitonin, serum lactate, DNI, and the Sequential Organ Failure Assessment (SOFA) scores were evaluated on days 0, 1, 3, and 7. We calculated the sensitivity, specificity, and diagnostic accuracy of CRP, procalcitonin, serum lactate, DNI, and SOFA scores. We used R software version 4.6.1 (R Foundation for Statistical Computing, Vienna, Austria) for data analysis. Results: We enrolled 937 patients with sepsis. Their median age was 56.0 (48.0-67.0) years. The study population was predominantly male (569, 60.73%). Two hundred thirty-four (24.97%) participants had SA-AKI. The median WBC counts among participants without and with AKI on day 0 were 18.5 (16.9-20.7) x 10⁹/L and 20.4 (18.4-22.8) x 10⁹/L, respectively (p < 0.001). Their median baseline serum creatinine values were 1.05 (0.96-1.12) mg/dL and 1.06 (0.97-1.16) mg/dL, respectively (p = 0.063). The corresponding median baseline DNI values were 8.0 (7.1-8.6)% and 9.1 (8.2-10.2)%, respectively (p < 0.001). On day 7, the DNI values were 7.0 (6.4-7.7)% and 8.4 (7.6-9.0)%, respectively (p < 0.001). Their median CRP values were 17.1 (14.4-20.9) mg/dL and 26.2 (22.6-30.2) mg/dL, respectively (p < 0.001). The median procalcitonin values were 1.98 (1.68-2.34) ng/mL and 2.11 (1.91-2.36) ng/mL, respectively (p < 0.001). The median serum lactate values were 2.6 (2.0-3.2) mmol/L and 2.6 (1.9-4.6) mmol/L, respectively (p = 0.004). All these parameters decreased over time. The sensitivity values for CRP, DNI, procalcitonin, serum lactate, and SOFA scores were 0.799, 0.594, 0.880, 0.308, and 0.026, respectively. Their specificity values were 0.764, 0.804, 0.351, 0.920, and 0.982, respectively. The corresponding diagnostic accuracy values were 0.773, 0.752, 0.483, 0.767, and 0.743, respectively. The AUCs for CRP, DNI, procalcitonin, serum lactate, and SOFA scores were 0.855 (0.831-0.880), 0.753 (0.716-0.790), 0.593 (0.556-0.630), 0.562 (0.515-0.610), and 0.436 (0.393-0.479), respectively. Conclusions: DNI values were significantly lower in patients with sepsis without AKI than in those with AKI. The diagnostic accuracy of DNI was comparable to CRP. Our results show that baseline DNI is a potential marker of SA-AKI, which can predict downstream risks of renal impairment in sepsis.

## Introduction

A dysregulated host reaction to an infection promotes sepsis, which often leads to a systemic inflammatory response, multiple organ dysfunction, and death [1-3]. Compared with high-income countries, low- and middle-income countries have a higher incidence of sepsis [4]. Sepsis-associated acute kidney injury (SA-AKI) increases the risk of chronic renal disease and cardiovascular events [5-7]. According to recent studies, the sepsis-related mortality rate in India is 28-36% [8,9].

According to the Sepsis-3 criteria [10], sepsis is defined as life-threatening organ dysfunction caused by a dysregulated host response to infection. The Sequential Organ Failure Assessment (SOFA) score [11] quantifies organ dysfunction. False-negative blood culture reports lead to delayed diagnosis and management of sepsis, SA-AKI, prolonged hospitalization, increased intensive care unit (ICU) admissions, morbidity, and mortality [12-14]. SA-AKI causes renal tubular epithelial cell damage, abnormalities in the coagulation cascade, and endothelial dysfunction [6,15,16]. Recent studies have suggested renal tubular cell death as the primary pathological manifestation of SA-AKI [17,18].

Numerous biomarkers, such as C-reactive protein (CRP), procalcitonin, and serum lactate, are being explored for early detection of SA-AKI [19-21]. CRP, an acute-phase reactant, is elevated in infectious, autoimmune, and inflammatory conditions [19,20]. Procalcitonin is useful in systemic inflammatory reactions and bacterial and viral infections [19,20]. It is also elevated in noninfectious inflammatory conditions, such as burns, trauma, or significant operations, which reduces its specificity in critically ill patients [14,19]. Elevated serum lactate levels point to tissue hypoxia, though it is not a specific biomarker of infection [14,21]. Furthermore, these biomarkers are relatively expensive, which limits their widespread use in developing countries [14,19-21]. Therefore, indices like immature granulocyte percentage, immature reticulocyte fraction, immature platelet fraction, systemic immune-inflammation index, and delta neutrophil index (DNI) are being assessed for early detection of sepsis and SA-AKI [14,22-24].

The DNI is computed by subtracting the percentage of polymorphonuclear leukocytes (PMN) from myeloperoxidase (MPO)-reactive cells (i.e., neutrophils and eosinophils) [24,25]. An automatic hematology analyzer, the ADVIA® 2120i (Siemens Healthcare Diagnostics, USA), provides the DNI value using the MPO and nuclear lobularity channels [24,25]. DNI can be used as a potential marker for predicting mortality in patients with sepsis and SA-AKI [24,26]. There is a dearth of literature regarding the predictive role of DNI in SA-AKI patients. Hence, we planned this study to evaluate the diagnostic accuracy of DNI in predicting SA-AKI in patients with sepsis and to compare it with that of CRP, procalcitonin, serum lactate, and the SOFA score.

## Materials and methods

We conducted this cross-sectional study from March 2024 to February 2026 at KIMS, Bhubaneswar, India. The Institutional Ethics Committee of Kalinga Institute of Medical Sciences granted ethics approval for this study (approval number KIIT/KIMS/IEC/1541/2024, dated 06.02.2024) prior to patient recruitment.

Study criteria

We enrolled all male and female adult patients with sepsis from our medicine ICU during the study period who met the Sepsis-3 criteria [10]. We excluded patients with any malignancy, myelodysplastic syndrome, autoimmune disease requiring immunosuppression, immunocompromised state, ongoing granulocyte colony-stimulating factor therapy, blood products, antibiotics (prophylactic or therapeutic), anticoagulants, antiplatelets, steroids, or anti-cancer drugs. Pregnant patients and patients with acute stroke, myocardial infarction, or upper GI bleeding were excluded. We also excluded those who were referred from other hospitals, were diagnosed with sepsis before ICU admission, died within 24 hours of sepsis diagnosis/onset, or stayed for less than seven days. Patients with a history of renal transplant, pre-existing renal disease, or baseline estimated glomerular filtration rate (eGFR) <60 mL/min/1.73 m² were also excluded.

Procedure

We noted the age, sex, marital status, and socioeconomic status of the participants from their case sheets. We used the Kuppuswamy classification [27] to assign participants to socioeconomic classes. We also noted whether participants had comorbidities (e.g., T2DM and hypertension). After sepsis was confirmed, we recorded the following parameters: white blood cell (WBC) count, serum creatinine, eGFR, SOFA score, CRP, procalcitonin, and serum lactate. We computed DNI values for the participants using an automated hematology analyzer, the ADVIA® 2120i (Siemens Healthcare Diagnostics, USA). It provided the proportion of neutrophils, eosinophils, and PMN in blood samples. The DNI value was calculated using the following formula: DNI (%) = neutrophil (%) + eosinophil (%) - PMN (%) [24,25]. These indices and parameters were evaluated again on days 1, 3, and 7. We analyzed the trends in all parameters (noted on days 0, 1, 3, and 7). We evaluated the sensitivity, specificity, threshold values, and diagnostic accuracy of CRP, DNI, procalcitonin, serum lactate, and SOFA scores for predicting AKI among participants. We assessed AKI status as per the Kidney Disease Improving Global Outcomes (KDIGO) criteria [28]. AKI was diagnosed if a participant met any of the following criteria: a rise in serum creatinine by ≥0.3 mg/dL within 48 hours, an increase in serum creatinine up to ≥1.5 times the baseline value within seven days, or a reduction in urine output by ≤0.5 mL/kg/hr for ≥6 consecutive hours [28].

Statistical analysis

For this cross-sectional study, non-probability consecutive sampling was used. Using the Shapiro-Wilk test, we assessed the normality of the data distribution. Continuous and categorical data were presented as medians with interquartile ranges (IQRs) and frequencies with proportions, respectively. The Wilcoxon rank-sum test was used for continuous data, and W-values were computed. No statistical comparisons were required for the categorical data. Hence, the chi-square test was not done. The receiver operating characteristic (ROC) curve analysis was performed for baseline CRP, DNI, procalcitonin, serum lactate, and SOFA scores. The area under the curve (AUC) was evaluated with a 95% confidence interval (CI). We used R software version 4.6.1 (R Foundation for Statistical Computing, Vienna, Austria) [29] to analyze the collected data. A p-value < 0.05 was considered statistically significant.

## Results

**Figure 1:**
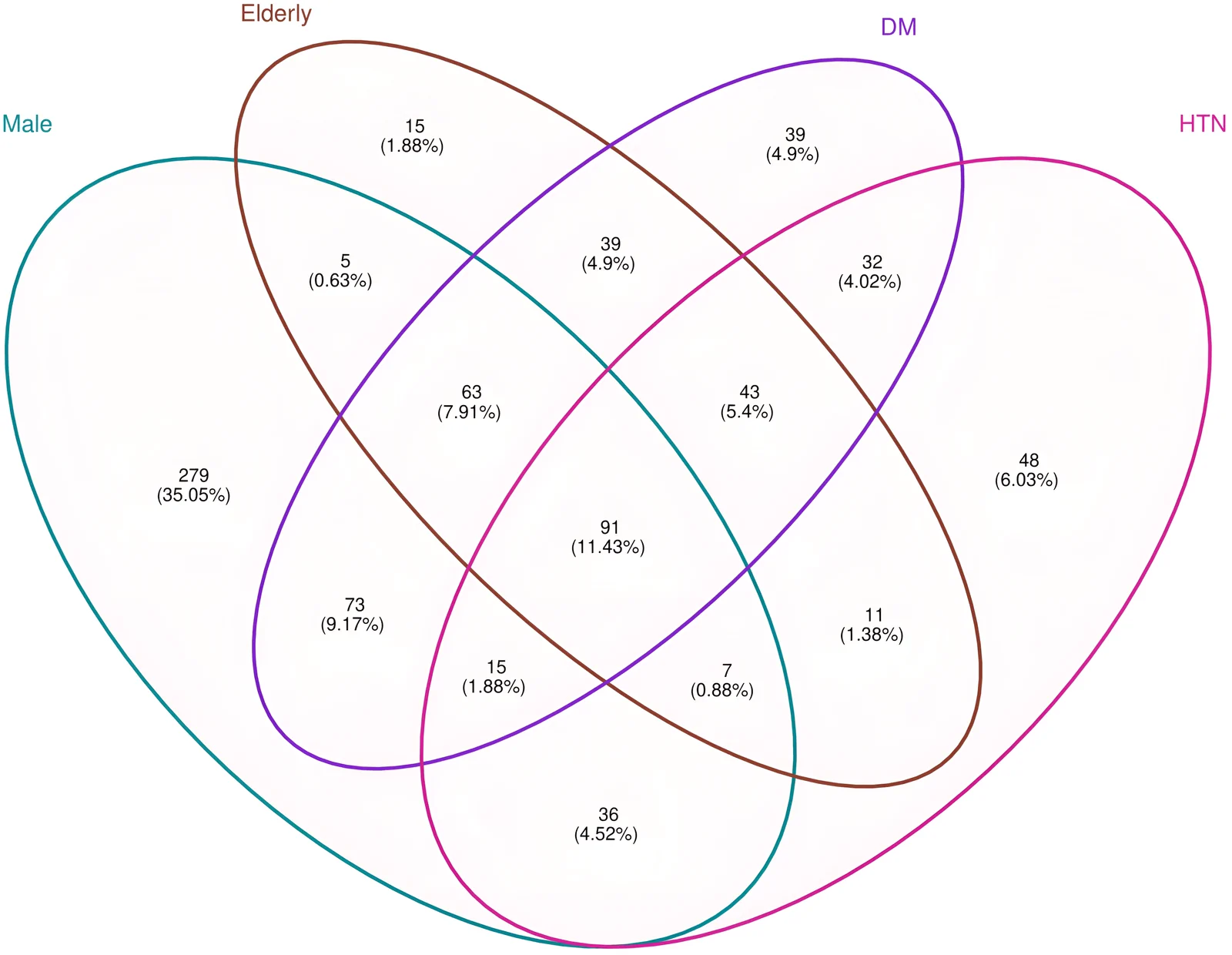
Distribution of the study population The Venn diagram shows the number and percentage of elderly male participants with diabetes and hypertension. The elderly group refers to those aged >65 years. DM: type 2 diabetes mellitus, HTN: hypertension

During the study period, a total of 1,973 patients hospitalized in the medicine ICU had sepsis. Three hundred forty-nine (17.69%) patients had sepsis before their admission. The case sheets of 687 (34.82%) patients were incomplete. The remaining 937 (47.49%) patients were assessed in this study. Table 1 shows the sociodemographic details of those 937 participants. The median age of the study population was 56.0 (48.0-67.0) years. Of them, 569 (60.73%) were male; 234 (24.97%) participants had AKI. The Venn diagram in Figure 1 shows the distribution of the study population by gender, age group, and the presence of T2DM and hypertension.

**Table 1:** Sociodemographic parameters of the study population (n = 937) Continuous and categorical data were expressed as medians with IQRs and counts with percentages, respectively. T2DM: type 2 diabetes mellitus, AKI: acute kidney injury, IQR: interquartile range

Parameters	Value
Age (years)	56.0 (48.0-67.0)
Elderly (age > 65 years)	274 (29.24%)
Male patients	569 (60.73%)
Marital status	
Married	860 (91.78%)
Unmarried	41 (4.38%)
Divorced/widowed	36 (3.84%)
Socioeconomic status	
Low	291 (31.06%)
Lower middle	604 (64.46%)
Upper middle	42 (4.48%)
T2DM	395 (42.16%)
Hypertension	283 (30.20%)
AKI	234 (24.97%)

**Figure 2:**
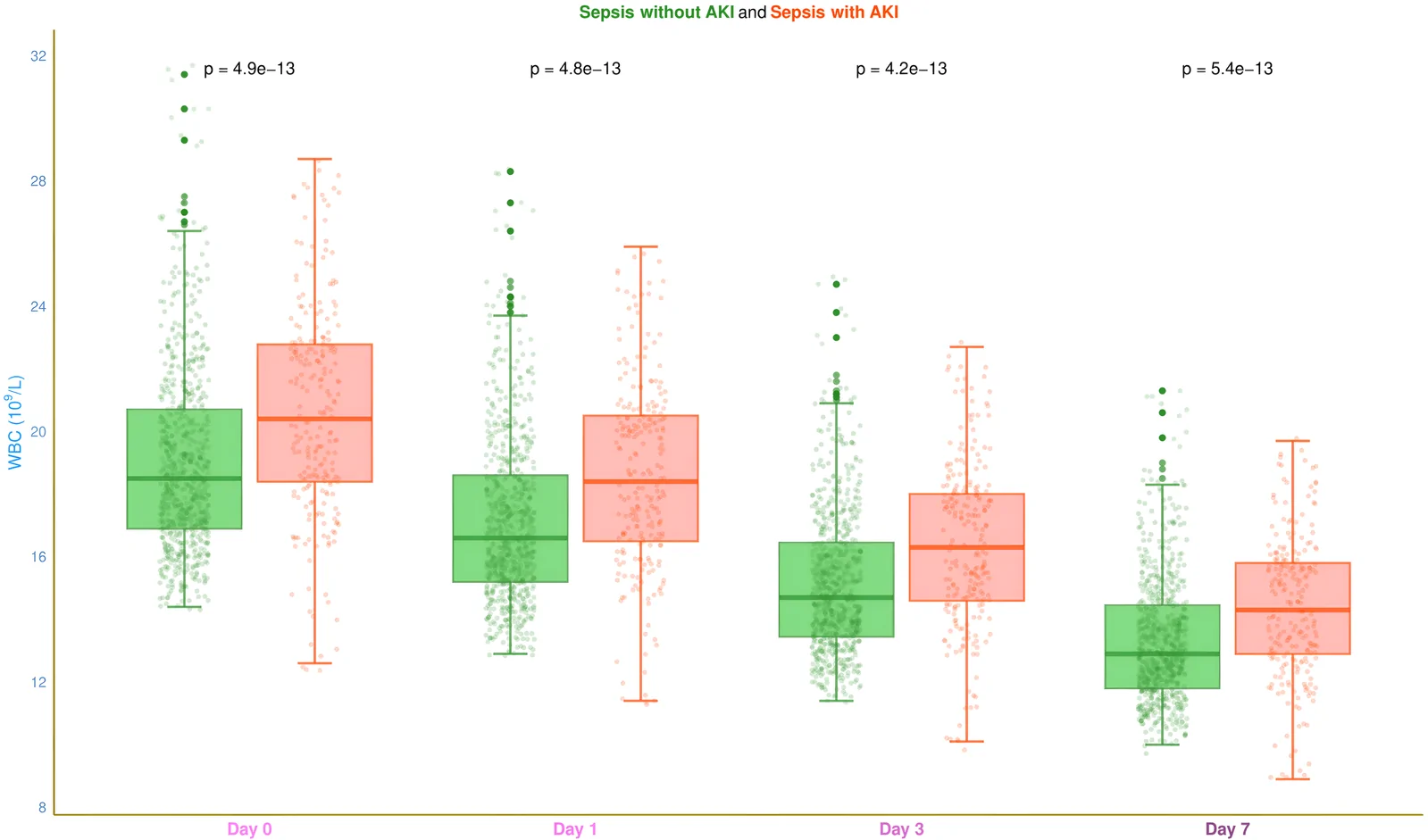
WBC counts of the study population at different time points The box-and-whisker and jitter plots show the WBC counts (×10⁹/L) of participants without and with AKI at different time points. We assessed these values using the Wilcoxon rank-sum test. Statistical significance was set at p < 0.05. AKI: acute kidney injury, WBC: white blood cell

Figure 2 and Table 2 show the WBC counts of the study population. The median WBC counts of participants without and with AKI on day 0 were 18.5 (16.9-20.7) x 10⁹/L and 20.4 (18.4-22.8) x 10⁹/L, respectively (p < 0.001). On day 7, the WBC counts were 12.9 (11.8-14.5) x 10⁹/L and 14.3 (12.9-15.8) x 10⁹/L, respectively (p < 0.001).

**Table 2:** WBC counts of the study population at different time points The WBC counts (×10⁹/L) were presented as median (IQR). We assessed these values using the Wilcoxon rank-sum test and calculated the W-values. Statistical significance was set at p < 0.05. AKI: acute kidney injury, IQR: interquartile range, WBC: white blood cell

Time point	Participants without AKI	Participants with AKI	W-value	p-value
Day 0	18.5 (16.9-20.7)	20.4 (18.4-22.8)	439453	<0.001
Day 1	16.6 (15.2-18.6)	18.4 (16.5-20.5)	346209	<0.001
Day 3	14.7 (13.5-16.5)	16.3 (14.6-18.0)	386716	<0.001
Day 7	12.9 (11.8-14.5)	14.3 (12.9-15.8)	367284	<0.001

**Figure 3:**
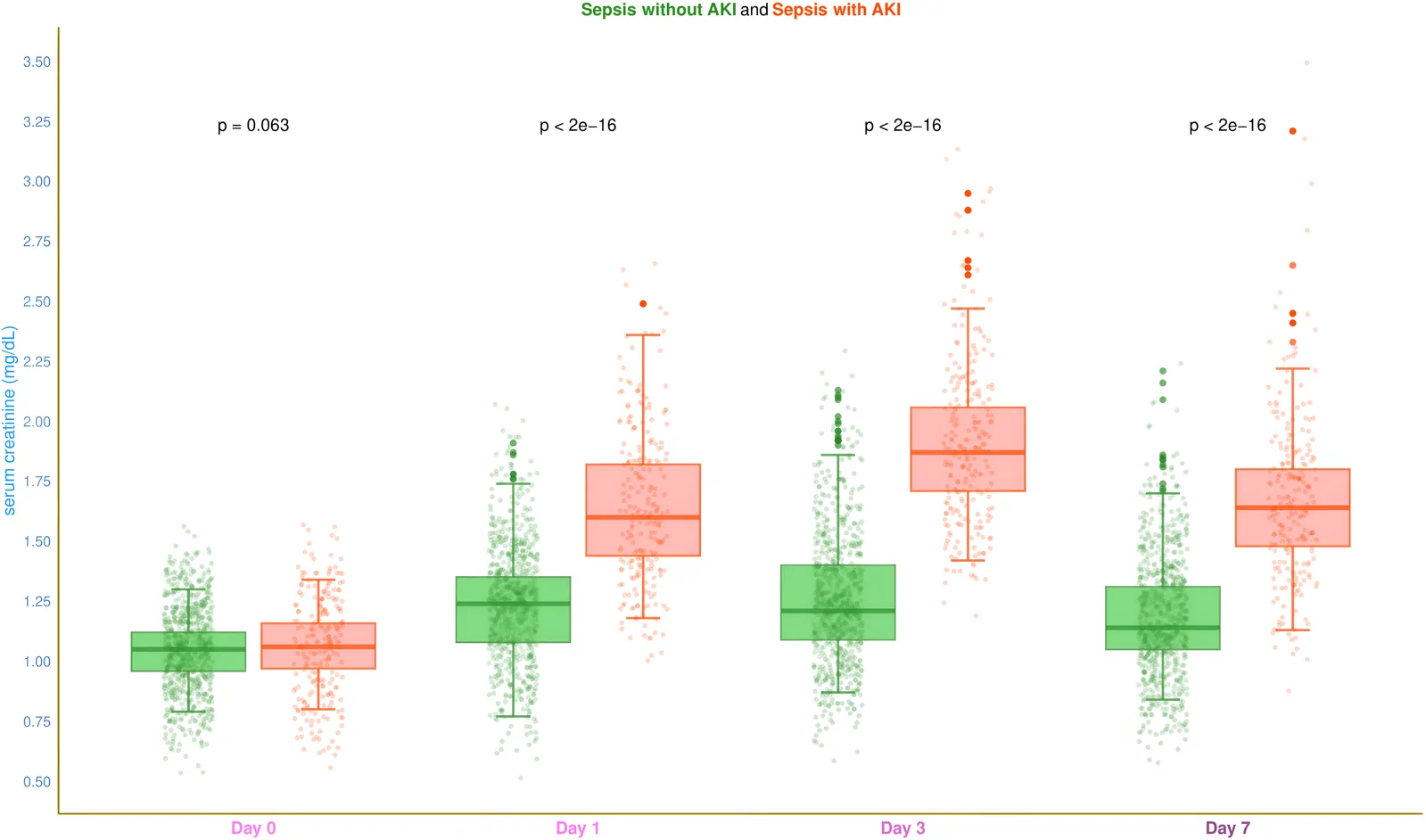
Serum creatinine of the study population at different time points The box-and-whisker and jitter plots show serum creatinine values (mg/dL) for participants without and with AKI at different time points. We assessed these values using the Wilcoxon rank-sum test. Statistical significance was set at p < 0.05. AKI: acute kidney injury

Figure 3 and Table 3 show the serum creatinine values of the study population. The median serum creatinine values of the participants without and with AKI on day 0 were 1.05 (0.96-1.12) mg/dL and 1.06 (0.97-1.16) mg/dL, respectively (p = 0.063). On day 7, the serum creatinine values were 1.14 (1.05-1.31) mg/dL and 1.64 (1.48-1.80) mg/dL, respectively (p < 0.001).

**Table 3:** Serum creatinine of the study population at different time points Serum creatinine values (mg/dL) were presented as median (IQR). We assessed these values using the Wilcoxon rank-sum test and calculated the W-values. Statistical significance was set at p < 0.05. AKI: acute kidney injury, IQR: interquartile range

Time point	Participants without AKI	Participants with AKI	W-value	p-value
Day 0	1.05 (0.96-1.12)	1.06 (0.97-1.16)	322981	0.063
Day 1	1.24 (1.08-1.35)	1.60 (1.44-1.82)	401714	<0.001
Day 3	1.21 (1.09-1.40)	1.87 (1.71-2.06)	397217	<0.001
Day 7	1.14 (1.05-1.31)	1.64 (1.48-1.80)	400342	<0.001

**Figure 4:**
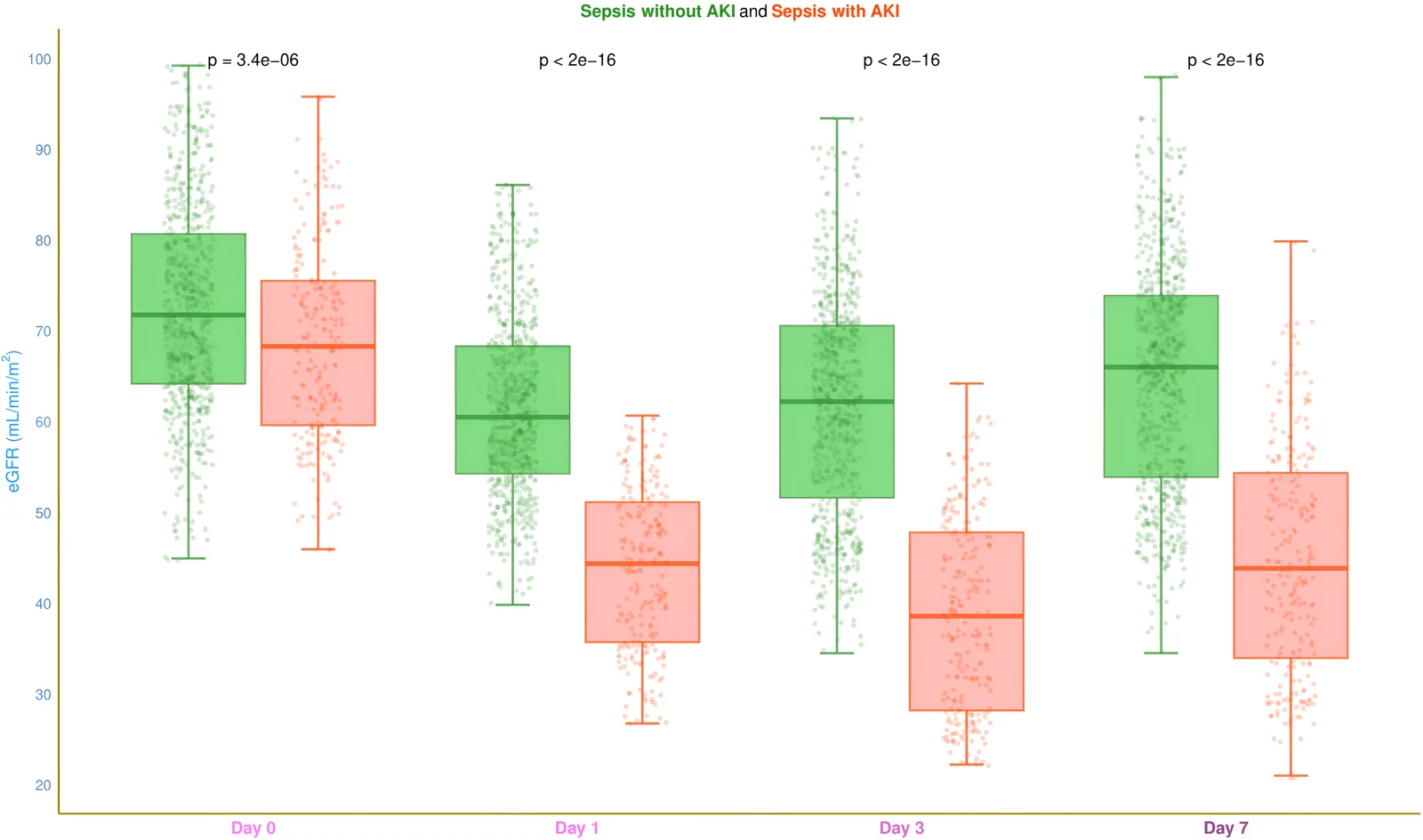
eGFR of the study population at different time points The box-and-whisker and jitter plots show eGFR values (mL/min/1.73 m²) for participants without and with AKI at different time points. We assessed these values using the Wilcoxon rank-sum test. Statistical significance was set at p < 0.05. eGFR: estimated glomerular filtration rate, AKI: acute kidney injury

Figure 4 and Table 4 show the eGFR values of the study population. The median eGFR values of the participants without and with AKI on day 0 were 71.80 (64.24-80.71) mL/min/1.73 m² and 68.33 (59.66-75.55) mL/min/1.73 m², respectively (p < 0.001). On day 7, the eGFR values were 66.06 (53.96-73.90) mL/min/1.73 m² and 43.87 (34.00-54.37) mL/min/1.73 m², respectively (p < 0.001).

**Table 4:** eGFR of the study population at different time points The eGFR values (mL/min/1.73 m²) were presented as median (IQR). We assessed these values using the Wilcoxon rank-sum test and calculated the W-values. Statistical significance was set at p < 0.05. eGFR: estimated glomerular filtration rate, AKI: acute kidney injury, IQR: interquartile range

Time point	Participants without AKI	Participants with AKI	W-value	p-value
Day 0	71.80 (64.24-80.71)	68.33 (59.66-75.55)	332198	<0.001
Day 1	60.54 (54.33-68.35)	44.40 (35.76-51.15)	399803	<0.001
Day 3	62.25 (51.68-70.59)	38.61 (28.22-47.83)	396105	<0.001
Day 7	66.06 (53.96-73.90)	43.87 (34.00-54.37)	402540	<0.001

**Figure 5:**
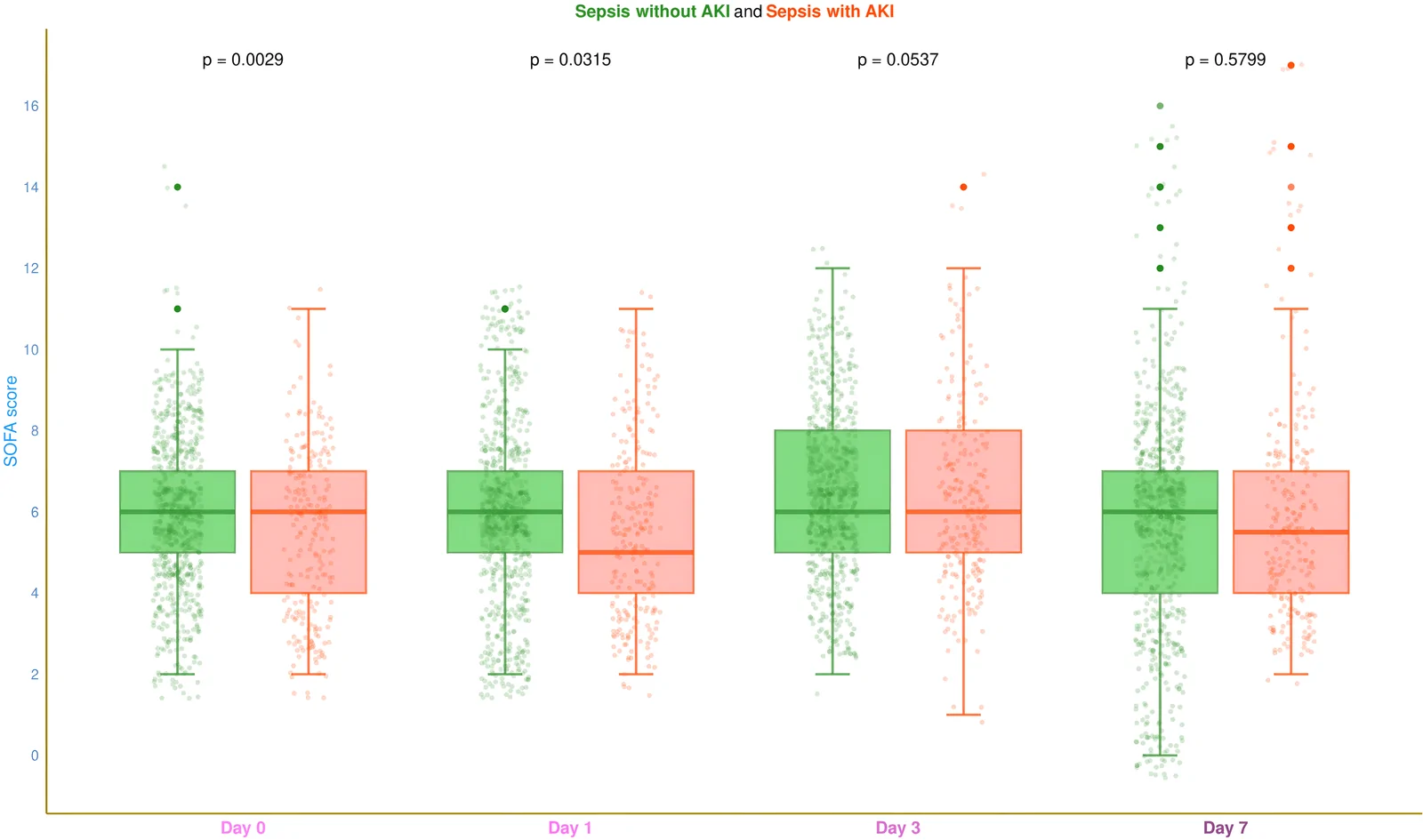
SOFA scores of the study population at different time points The box-and-whisker and jitter plots show the SOFA scores of participants without and with AKI at different time points. We assessed these values using the Wilcoxon rank-sum test. Statistical significance was set at p < 0.05. SOFA: Sequential Organ Failure Assessment, AKI: acute kidney injury

Figure 5 and Table 5 show the SOFA scores of the study population. The median SOFA scores of the participants without and with AKI on day 0 were 6.0 (5.0-7.0) and 6.0 (4.0-7.0), respectively (p = 0.003). On day 7, the SOFA scores were 6.0 (4.0-7.0) and 5.5 (4.0-7.0), respectively (p = 0.580).

**Table 5:** SOFA scores of the study population at different time points The SOFA scores were presented as median (IQR). We assessed these values using the Wilcoxon rank-sum test and calculated the W-values. Statistical significance was set at p < 0.05. SOFA: Sequential Organ Failure Assessment, AKI: acute kidney injury, IQR: interquartile range

Time point	Participants without AKI	Participants with AKI	W-value	p-value
Day 0	6.0 (5.0-7.0)	6.0 (4.0-7.0)	318996	0.003
Day 1	6.0 (5.0-7.0)	5.0 (4.0-7.0)	321974	0.032
Day 3	6.0 (5.0-8.0)	6.0 (5.0-8.0)	322726	0.054
Day 7	6.0 (4.0-7.0)	5.5 (4.0-7.0)	327682	0.580

**Figure 6:**
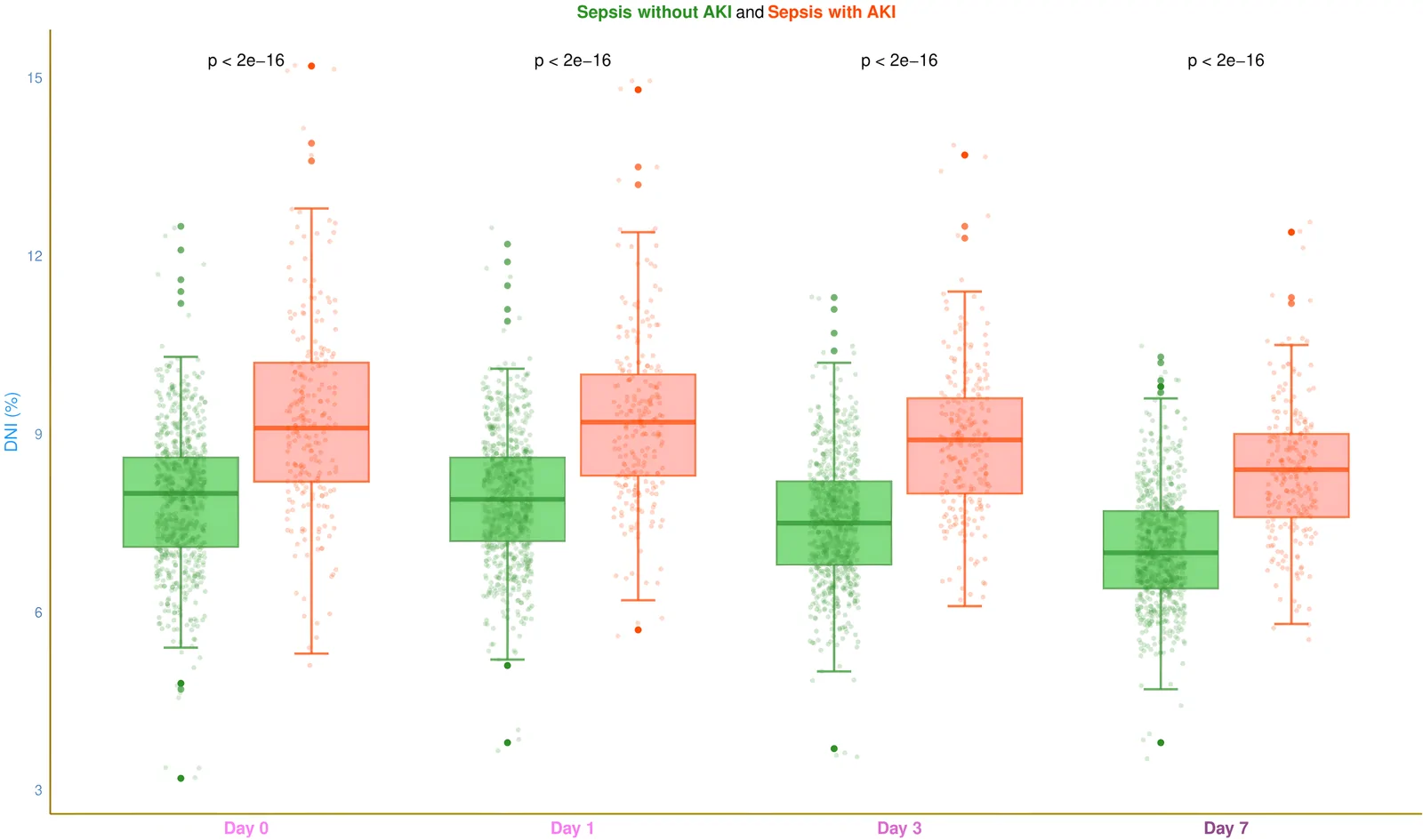
DNI values of the study population at different time points The box-and-whisker and jitter plots show the DNI values (%) for participants without and with AKI at different time points. We assessed these values using the Wilcoxon rank-sum test. Statistical significance was set at p < 0.05. DNI: delta neutrophil index, AKI: acute kidney injury

Figure 6 and Table 6 show the DNI values of the study population. The median DNI values for participants without and with AKI on day 0 were 8.0 (7.1-8.6)% and 9.1 (8.2-10.2)%, respectively (p < 0.001). On day 7, the DNI values were 7.0 (6.4-7.7)% and 8.4 (7.6-9.0)%, respectively (p < 0.001).

**Table 6:** DNI values of the study population at different time points The DNI values (%) were presented as median (IQR). We assessed these values using the Wilcoxon rank-sum test and calculated the W-values. Statistical significance was set at p < 0.05. DNI: delta neutrophil index, AKI: acute kidney injury, IQR: interquartile range

Time point	Participants without AKI	Participants with AKI	W-value	p-value
Day 0	8.0 (7.1-8.6)	9.1 (8.2-10.2)	416298	<0.001
Day 1	7.9 (7.2-8.6)	9.2 (8.3-10.0)	406045	<0.001
Day 3	8.0 (7.1-8.6)	9.1 (8.2-10.2)	402665	<0.001
Day 7	7.0 (6.4-7.7)	8.4 (7.6-9.0)	412477	<0.001

**Figure 7:**
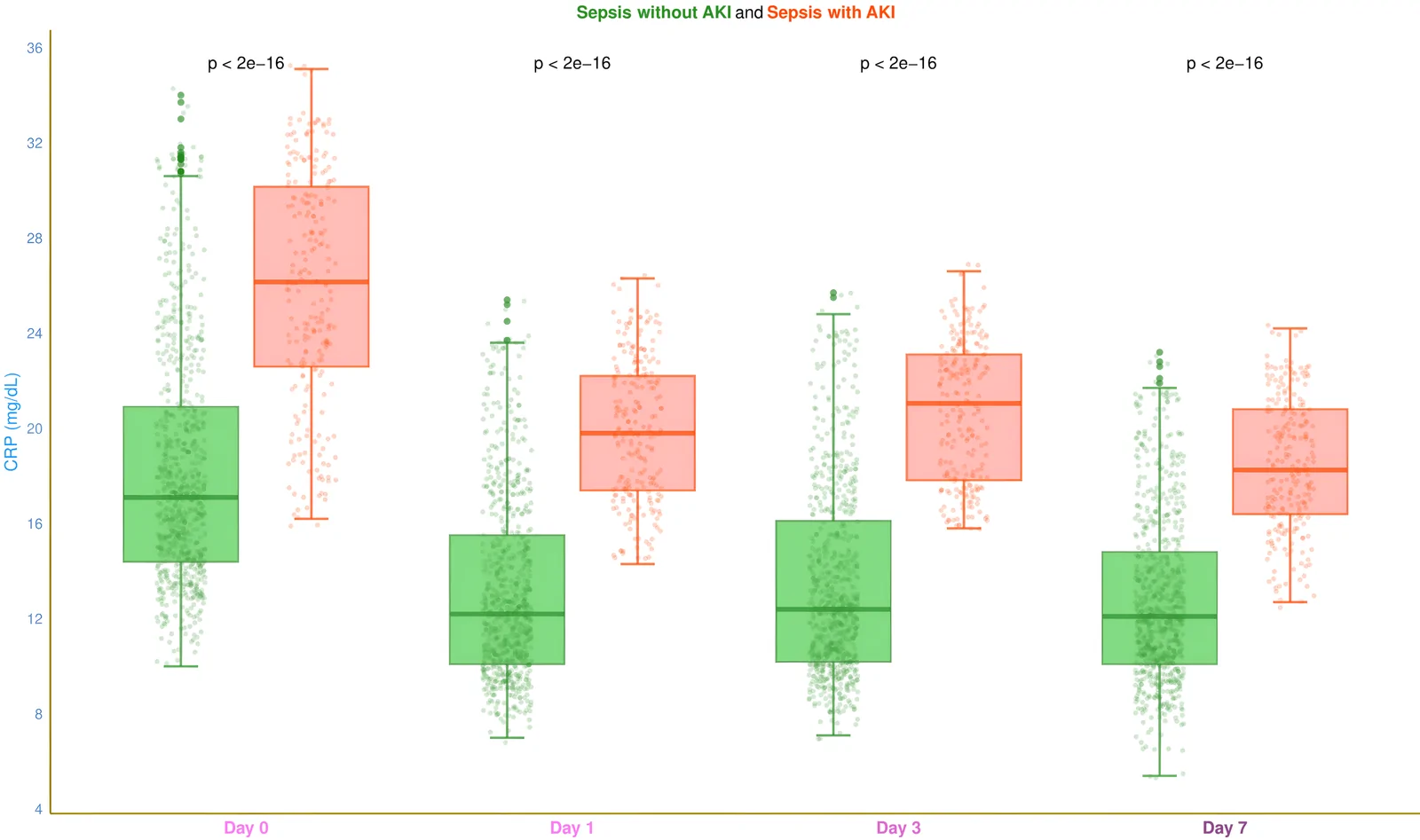
CRP values of the study population at different time points The box-and-whisker and jitter plots show CRP values (mg/dL) for participants without and with AKI at different time points. We assessed these values using the Wilcoxon rank-sum test. Statistical significance was set at p < 0.05. CRP: C-reactive protein, AKI: acute kidney injury

Figure 7 and Table 7 show the CRP values of the study population. The median CRP values for participants without and with AKI on day 0 were 17.1 (14.4-20.9) mg/dL and 26.2 (22.6-30.2) mg/dL, respectively (p < 0.001). On day 7, the CRP values were 12.1 (10.1-14.8) mg/dL and 18.3 (16.4-20.8) mg/dL, respectively (p < 0.001).

**Table 7:** CRP values of the study population at different time points The CRP values (mg/dL) were presented as median (IQR). We assessed these values using the Wilcoxon rank-sum test and calculated the W-values. Statistical significance was set at p < 0.05. CRP: C-reactive protein, AKI: acute kidney injury, IQR: interquartile range

Time point	Participants without AKI	Participants with AKI	W-value	p-value
Day 0	17.1 (14.4-20.9)	26.2 (22.6-30.2)	301298	<0.001
Day 1	12.2 (10.1-15.5)	19.8 (17.4-22.2)	301648	<0.001
Day 3	12.4 (10.2-16.1)	21.1 (17.8-23.1)	300679	<0.001
Day 7	12.1 (10.1-14.8)	18.3 (16.4-20.8)	310024	<0.001

**Figure 8:**
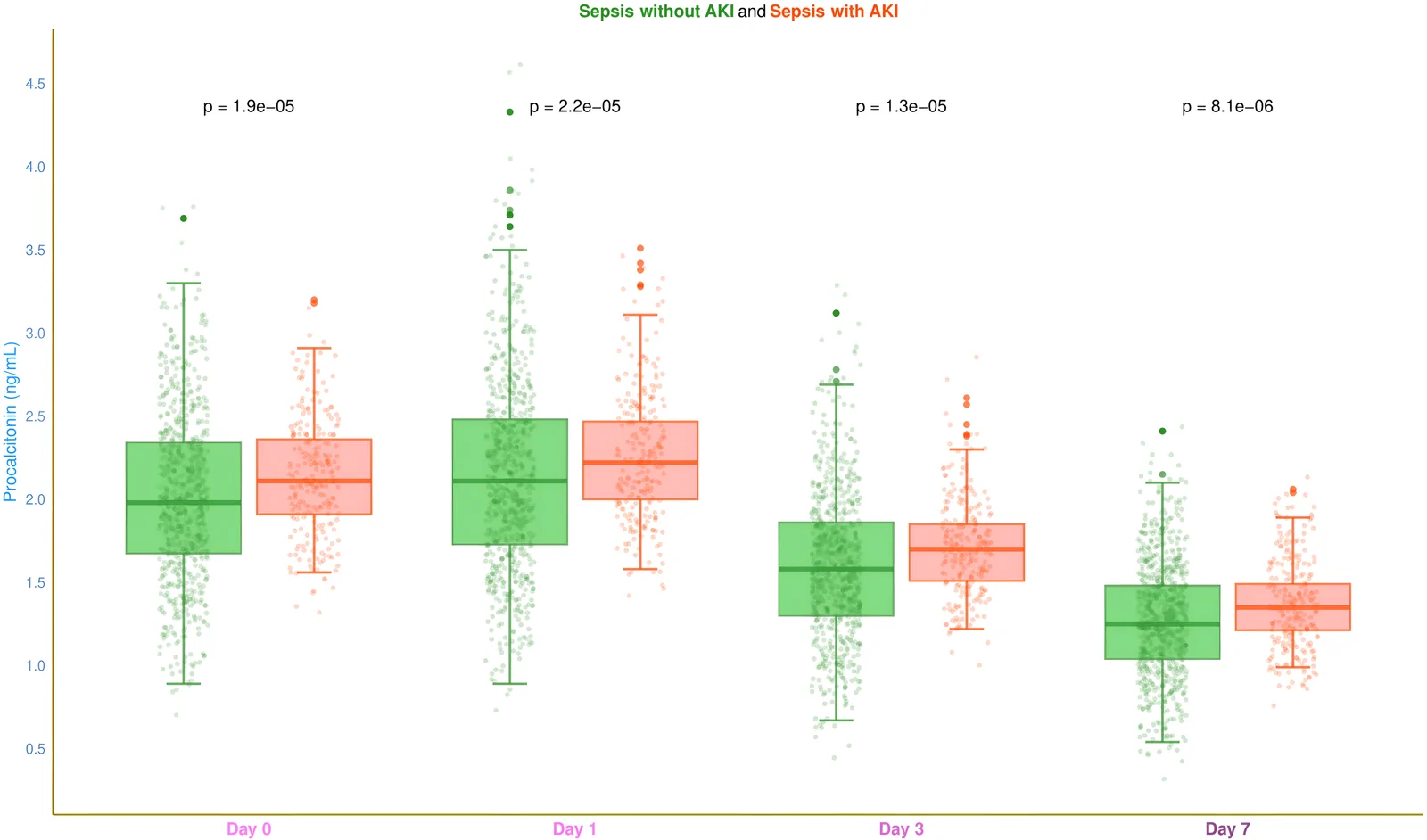
Procalcitonin values of the study population at different time points The box-and-whisker and jitter plots show procalcitonin values (ng/mL) for participants without and with AKI at different time points. We assessed these values using the Wilcoxon rank-sum test. Statistical significance was set at p < 0.05. AKI: acute kidney injury

Figure 8 and Table 8 show the procalcitonin values of the study population. The median procalcitonin values for participants without and with AKI on day 0 were 1.98 (1.68-2.34) ng/mL and 2.11 (1.91-2.36) ng/mL, respectively (p < 0.001). On day 7, the procalcitonin values were 1.25 (1.04-1.48) ng/mL and 1.35 (1.21-1.49) ng/mL, respectively (p < 0.001).

**Table 8:** Procalcitonin values of the study population at different time points The procalcitonin values (ng/mL) were presented as median (IQR). We assessed these values using the Wilcoxon rank-sum test and calculated the W-values. Statistical significance was set at p < 0.05. AKI: acute kidney injury, IQR: interquartile range

Time point	Participants without AKI	Participants with AKI	W-value	p-value
Day 0	1.98 (1.68-2.34)	2.11 (1.91-2.36)	346718	<0.001
Day 1	2.11 (1.73-2.48)	2.22 (2.00-2.48)	356382	<0.001
Day 3	1.58 (1.30-1.86)	1.70 (1.51-1.85)	341652	<0.001
Day 7	1.25 (1.04-1.48)	1.35 (1.21-1.49)	354289	<0.001

**Figure 9:**
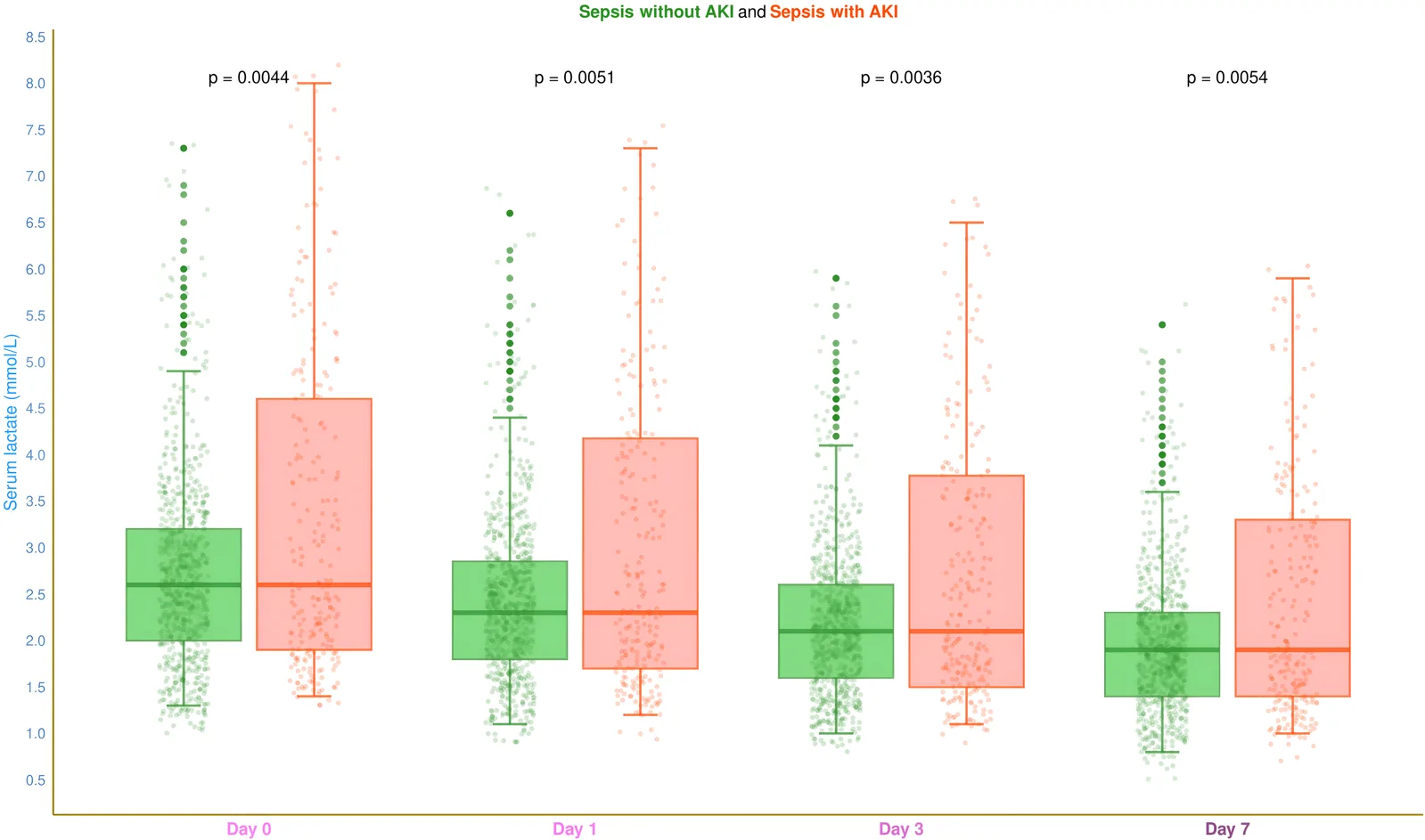
Serum lactate values of the study population at different time points The box-and-whisker and jitter plots show serum lactate values (mmol/L) for participants without and with AKI at different time points. We assessed these values using the Wilcoxon rank-sum test. Statistical significance was set at p < 0.05. AKI: acute kidney injury

Figure 9 and Table 9 show the serum lactate values of the study population. The median serum lactate values for participants without and with AKI on day 0 were 2.6 (2.0-3.2) mmol/L and 2.6 (1.9-4.6) mmol/L, respectively (p = 0.004). On day 7, the serum lactate values were 1.9 (1.4-2.3) mmol/L and 1.9 (1.4-3.3) mmol/L, respectively (p = 0.005).

**Table 9:** Serum lactate values of the study population at different time points Serum lactate values (mmol/L) were presented as median (IQR). We assessed these values using the Wilcoxon rank-sum test and calculated the W-values. Statistical significance was set at p < 0.05. AKI: acute kidney injury, IQR: interquartile range

Time point	Participants without AKI	Participants with AKI	W-value	p-value
Day 0	2.6 (2.0-3.2)	2.6 (1.9-4.6)	319318	0.004
Day 1	2.3 (1.8-2.9)	2.3 (1.7-4.2)	319668	0.005
Day 3	2.1 (1.6-2.6)	2.1 (1.5-3.8)	319467	0.004
Day 7	1.9 (1.4-2.3)	1.9 (1.4-3.3)	319718	0.005

**Figure 10:**
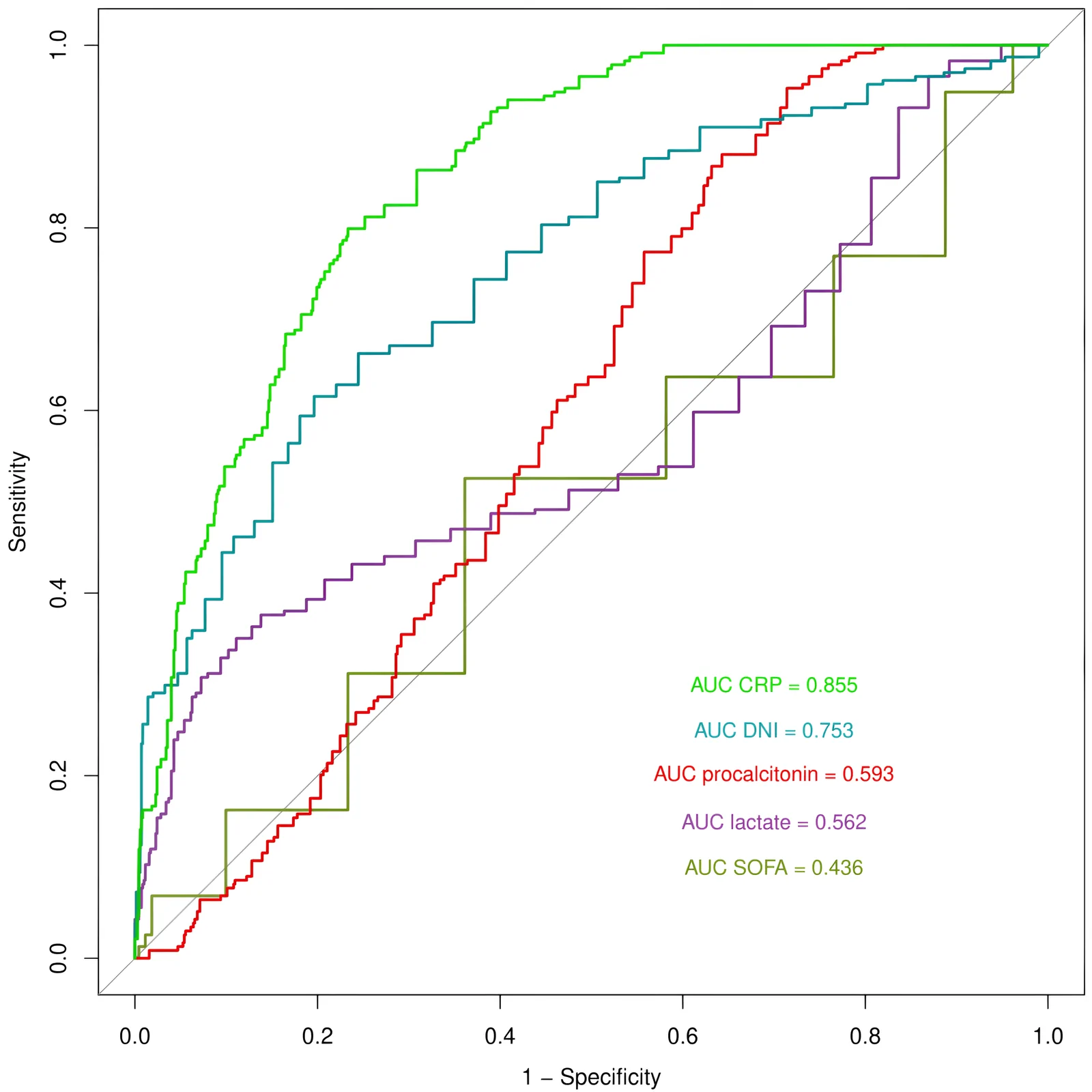
ROC curve for various indices The X- and Y-axes show 1-specificity and sensitivity values for baseline CRP, DNI, procalcitonin, serum lactate, and SOFA scores. The five ROC curves and their AUC values are shown in different colors. CRP: C-reactive protein, DNI: delta neutrophil index, SOFA: Sequential Organ Failure Assessment, ROC: receiver operating characteristic, AUC: area under the curve

Figure 10 shows the ROC curves for baseline CRP, DNI, procalcitonin, serum lactate, and SOFA scores. Table 10 shows the sensitivity, specificity, threshold value, diagnostic accuracy, and AUC of the ROC curve, along with their 95% CIs. The sensitivity values for CRP, DNI, procalcitonin, serum lactate, and SOFA scores were 0.799, 0.594, 0.880, 0.308, and 0.026, respectively. The corresponding specificity values were 0.764, 0.804, 0.351, 0.920, and 0.982, respectively. The threshold values were 21.35, 8.85, 1.81, 4.15, and 9.5, respectively. The diagnostic accuracy values of the five indices were 0.773, 0.752, 0.483, 0.767, and 0.743, respectively. The AUCs for CRP, DNI, procalcitonin, serum lactate, and SOFA scores were 0.855 (0.831-0.880), 0.753 (0.716-0.790), 0.593 (0.556-0.630), 0.562 (0.515-0.610), and 0.436 (0.393-0.479), respectively.

**Table 10:** Statistical comparison of various indices The ranges for sensitivity, specificity, diagnostic accuracy, and AUC for baseline CRP, DNI, procalcitonin, serum lactate, and SOFA scores were 0-1. CRP: C-reactive protein, DNI: delta neutrophil index, SOFA: Sequential Organ Failure Assessment, ROC: receiver operating characteristic, AUC: area under the curve, CI: confidence interval

Parameters	Sensitivity	Specificity	Diagnostic accuracy	Threshold value	AUC	95% CI for AUC
CRP	0.799	0.764	0.773	21.35	0.855	0.831-0.880
DNI	0.594	0.804	0.752	8.85	0.753	0.716-0.790
Procalcitonin	0.880	0.351	0.483	1.81	0.593	0.556-0.630
Lactate	0.308	0.920	0.767	4.15	0.562	0.515-0.610
SOFA	0.026	0.982	0.743	9.5	0.436	0.393-0.479

## Discussion

Our study evaluated WBC count, serum creatinine, eGFR, SOFA scores, DNI, CRP, procalcitonin, and serum lactate in participants without and with AKI on days 0, 1, 3, and 7. We calculated and compared the diagnostic accuracy of the SOFA score, DNI, CRP, procalcitonin, and serum lactate for predicting SA-AKI. We found that DNI and CRP had similar diagnostic accuracies. As CRP is a non-specific acute-phase reactant [14,19,20], we identified DNI as an emerging biomarker for SA-AKI. Sepsis and SA-AKI can lead to prolonged ICU stays and increased morbidity and mortality [6,15,22]. Timely and precise diagnosis of SA-AKI is necessary to improve outcomes [6,7,16,17,19].

Risk factors for sepsis in ICU patients include prolonged ICU stays, immunocompromised state, multiple indwelling catheters, catheter-related bloodstream infections, hospital-acquired infections (HAIs), and polypharmacy [2,3,8,9,30]. The hallmark of SA-AKI is a sudden deterioration in renal function, frequently triggered by a complex interplay among immunological, inflammatory, and hemodynamic factors [6,15-18]. Inflammatory mediators, including damage-associated molecular patterns (DAMPs) and pathogen-associated molecular patterns (PAMPs), are released into the intravascular space during sepsis and bind to immune cell surface receptors, namely Toll-like receptors (TLRs) [6,7]. This response then triggers a signaling cascade that produces pro-inflammatory cytokines. Furthermore, TLR-2 and TLR-4 are expressed by renal tubular epithelial cells [6,7]. As a result, filtration of PAMPs or DAMPs through the glomerulus triggers oxidative stress, the generation of reactive oxygen species (ROS), and mitochondrial damage [6,7,15,16,18].

Reduced perfusion and oxygenation of the renal medulla result from diversion of renal blood flow from the medulla to the cortex, thereby worsening kidney function [6,7]. Increased pro-inflammatory cytokines in sepsis may promote microthrombosis in renal capillaries [5,6]. Additionally, these vascular dysfunctions trigger ROS production, which worsens the epithelial barrier and ultimately produces an endothelial leak [6,7]. Lipopolysaccharide (LPS), a circulating endotoxin and component of the bacterial cell wall, can cause AKI by directly inducing apoptosis in renal tubular cells through the production of systemic cytokines [15,16]. Tumor necrosis factor-α and LPS both induce apoptosis in glomerular endothelial cells during gram-negative infection, leading to SA-AKI [6,15,16].

DNI was first developed to reflect immature granulocytes in circulating blood [24-26]. Mathematically, it can be regarded as the difference between the leukocyte fractions tested in the MPO and the nuclear lobularity channels [24,25]. In sepsis and SIRS, a left shift in neutrophils has been observed, reflecting an increase in immature granulocytes [24,26]. Bacterial infections, including sepsis, in ICU patients might cause an elevated WBC count [14,16,24]. Conversely, decreased bone marrow production and peripheral overconsumption and/or destruction in reaction to disseminated intravascular coagulation have been proposed as explanations for sepsis-associated leukopenia [24]. DNI offers an attractive alternative for predicting SA-AKI among sepsis patients in the ICU [16,24,26]. Prompt diagnosis of SA-AKI may be possible due to the early elevation of DNI, which reflects a real-time marrow reaction occurring prior to systemic organ damage [24,26].

Strengths and limitations

A strength of our study was the comparative analysis of SOFA scores, DNI, CRP, procalcitonin, and serum lactate values among sepsis patients in predicting SA-AKI. Our study also had a few limitations. First, data from adult patients enrolled in a specific department at a single center, subject to stringent criteria, cannot be generalized. Second, the analyses of the source of infection, duration of ICU stays, and progression of renal dysfunction were beyond the scope of this study. Third, the impacts of polypharmacy and comorbidities on the clinical parameters of sepsis patients were not assessed. Fourth, we could not perform risk stratification of patients as per DNI, which could have helped guide the selection/use of appropriate antibiotics.

## Conclusions

The sepsis patients without AKI had significantly lower DNI values than those with AKI. DNI showed diagnostic accuracy similar to that of CRP. Our findings demonstrate that baseline DNI is a reliable metric that captures key components, such as neutrophilic activation and early host immunological dysregulation, that appear to anticipate downstream risks of renal impairment in sepsis. We noticed rapid resolution of systemic inflammatory markers (e.g., DNI and CRP) in contrast to delayed and incomplete renal recovery. These findings support the role of non-hemodynamic pathways (e.g., microvascular dysfunction, neutrophil-mediated tubular stress, oxidative damage) in the pathophysiology of SA-AKI. We suggest prospective studies with larger sample sizes and longer follow-up periods to compare these parameters for predicting SA-AKI progression.
